# STAT3 operates as an inflammation-dependent transcriptional switch

**DOI:** 10.1101/2025.09.26.678857

**Published:** 2025-09-28

**Authors:** Simon Grassmann, Hyunu Kim, Christin Friedrich, Marine Pujol, Sherry Fan, Jennifer Zhang, Giorgi Beroshvili, Veit R. Buchholz, Georg Gasteiger, Joseph C. Sun

## Abstract

Signal transducer and activator of transcription 3 (STAT3) is a key regulator of immune cell function, but its role in lymphocytes remains incompletely understood. Here, we show that STAT3 has a context-dependent function in antiviral natural killer (NK) cells, either promoting or impairing adaptive NK cell responses dependent on the level of inflammation. STAT3 is recruited to distinct genomic sites under homeostatic versus inflammatory environments, where it drives different transcriptional programs. Through this re-localization, STAT3 regulates downstream transcription factors MYB and BLIMP-1 in an inflammation-dependent manner to shape NK cell differentiation under homeostasis and during infection. Thus, STAT3 acts as a transcriptional switch that integrates cytokine signals to control lymphocyte adaptation to different environments. This mechanism highlights how therapeutic interventions targeting STAT3 can result in different outcomes depending on the degree of inflammation.

**HIGHLIGHTS:**

STAT3 exerts a context-dependent role on adaptive NK cells during viral infection
STAT3 modulates IL-15 signaling by competing with STAT5 and regulating MYB
Homeostatic versus inflammatory cytokines relocate STAT3 to distinct genomic sites
Downstream transcription factors MYB and BLIMP-1 govern STAT3-dependent differentiation

## Full Text Availability

The license terms selected by the author(s) for this preprint version do not permit archiving in PMC. The full text is available from the preprint server.

